# First-Time Use of Diced Cartilage Glue Grafts for Post Trauma Orbital Rim Reconstruction

**DOI:** 10.1177/22925503251355976

**Published:** 2025-07-11

**Authors:** Akhil Nair, Mark McRae, Matthew McRae, Joseph Catapano, Blake Murphy

**Affiliations:** 1 12366 Temerty Faculty of Medicine, University of Toronto, Toronto, Ontario, Canada; 2 Division of Plastic and Reconstructive Surgery, University of Toronto, Toronto, Ontario, Canada; 3 Division of Plastic Surgery, Unity Health, 10071 St. Michael's Hospital, Toronto, Ontario, Canada

**Keywords:** orbital reconstruction, orbital augmentation, orbital contouring, diced cartilage graft, diced cartilage and fibrin glue, reconstruction orbitaire, augmentation orbitaire, lissage orbitaire, greffe de cartilage en dés, cartilage en dés et colle de fibrine

## Abstract

Diced cartilage glue graft is a technique where small pieces of diced cartilage are mixed with fibrin glue to form a malleable cohesive graft. This technique is routinely used in rhinoplasty or nasal dorsum augmentation. Here we present the case of a 52-year-old man who sustained craniofacial trauma and developed supraorbital irregularities following the primary reconstruction surgeries. We, for the first time, used diced glue graft technique to perform superomedial orbital rim reconstruction and contouring to resolve the irregularities. Based on our experience, this method can be successfully adapted for orbital rim reconstruction while achieving seamless contouring and enhanced aesthetic results.

## Résumé

La greffe de colle de cartilage en dés est une technique dans laquelle de petits fragments de cartilages coupés en dés sont mélangés à de la colle de fibrine pour former un greffon malléable et cohésif. Cette technique est régulièrement utilisée dans les rhinoplasties ou les augmentations de l’arête du nez. Nous présentons ici le cas d’un homme de 52 ans ayant subi un traumatisme craniofacial chez qui se sont développées des irrégularités supra-orbitaires après les chirurgies de reconstruction primaires. Nous avons utilisé pour la première fois la technique de greffe de colle et dés pour réaliser une reconstruction du rebord orbitaire supéromédial et pour retracer le contour en supprimant les irrégularités. D’après notre expérience, cette méthode peut être adaptée avec succès pour la reconstruction du rebord orbitaire tout en obtenant un lissage régulier et des résultats esthétiques améliorés.

## Case Presentation

**Figure 1.**
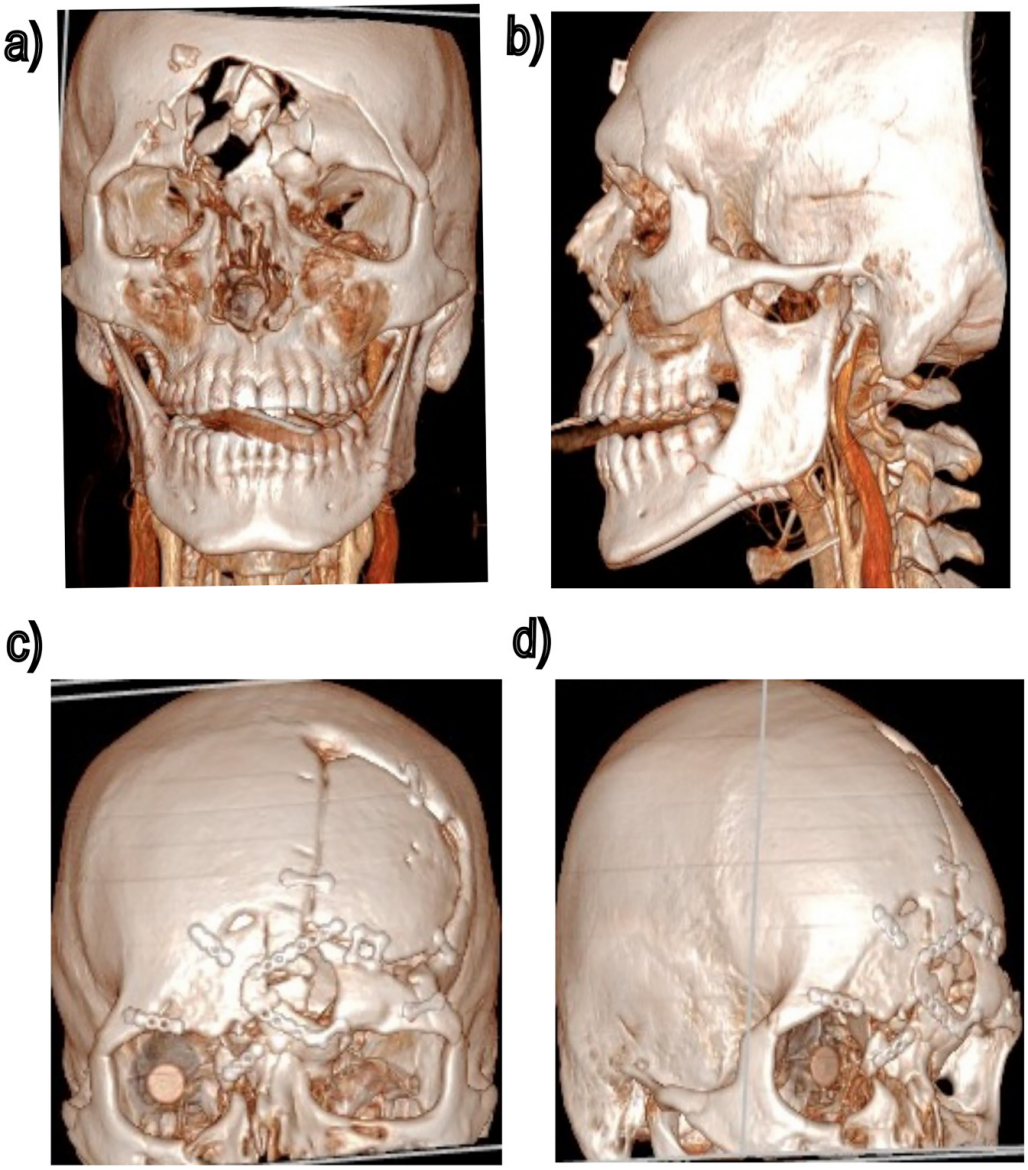
(a, b) Three-dimensional computed tomography (3D CT) scans of the primary injury. (c, d) 3D CT scans after calvarial reconstruction.

**Figure 2.**
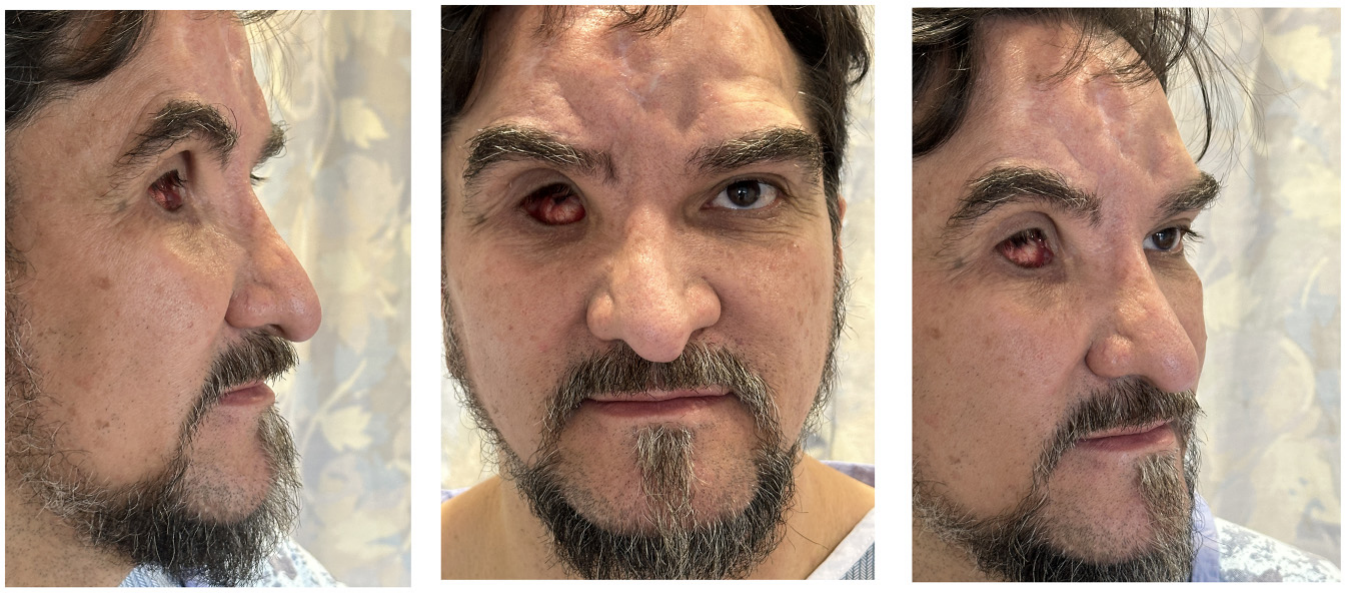
Preoperative pictures showing the orbital irregularities (with prosthesis removed).

A 52-year-old male presented with a blast injury associated with right-sided facial trauma and right eye vision loss (Figure 1a and b). Initial surgical intervention reconstructed the calvarium and the right orbit using salvaged bone fragments and a split cranial graft from the contralateral side, while also adding a right ocular prosthesis (Figure 1c and d). After 18 months, the patient presented with a concern of supraorbital rim irregularity and depression of the craniofacial skeleton underlying the soft tissue. The irregularity extended 2.5 cm (Figure 2) from the nasal radix along the right nasal sidewall to the medial orbital rim. The patient also had an irregular forehead contour from trauma to bone, soft tissue, and skin, along with nasal tip support loss due to nasal, nasoorbitoethmoid, and septal fractures.

## Operative Intervention

**Figure 3.**
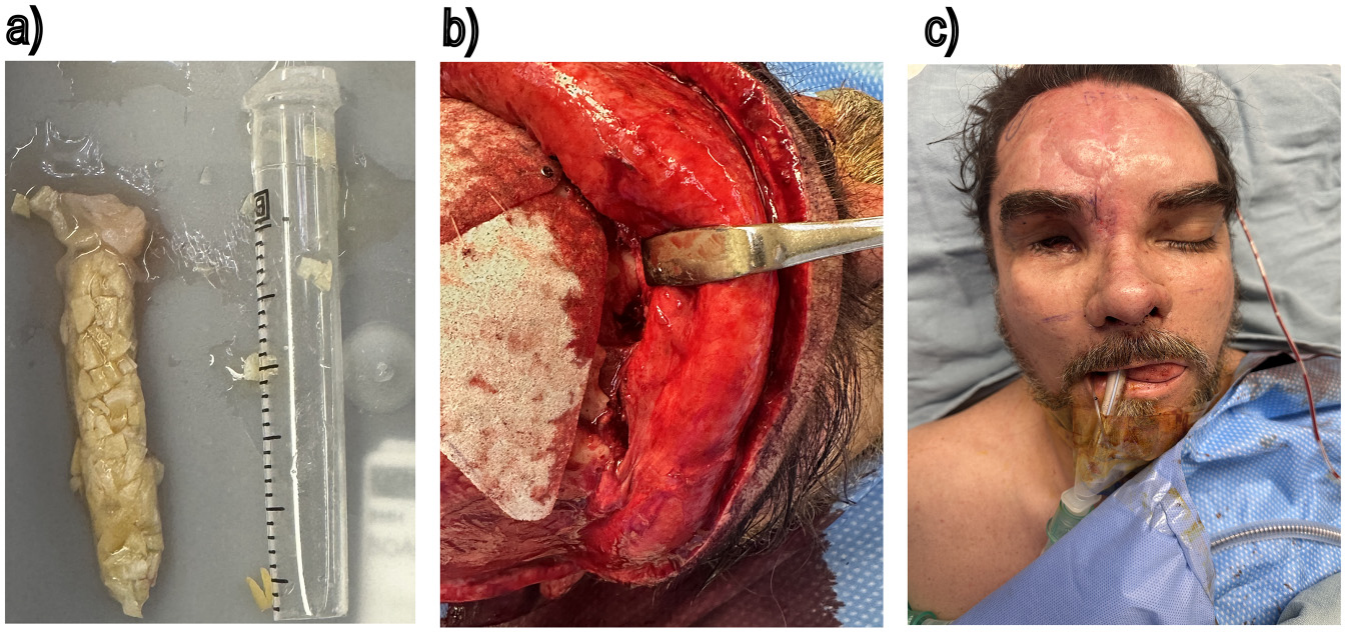
(a) Diced cartilage glue graft, (b) superomedial orbital osteotomy, and (c) intraoperative results.

**Figure 4.**
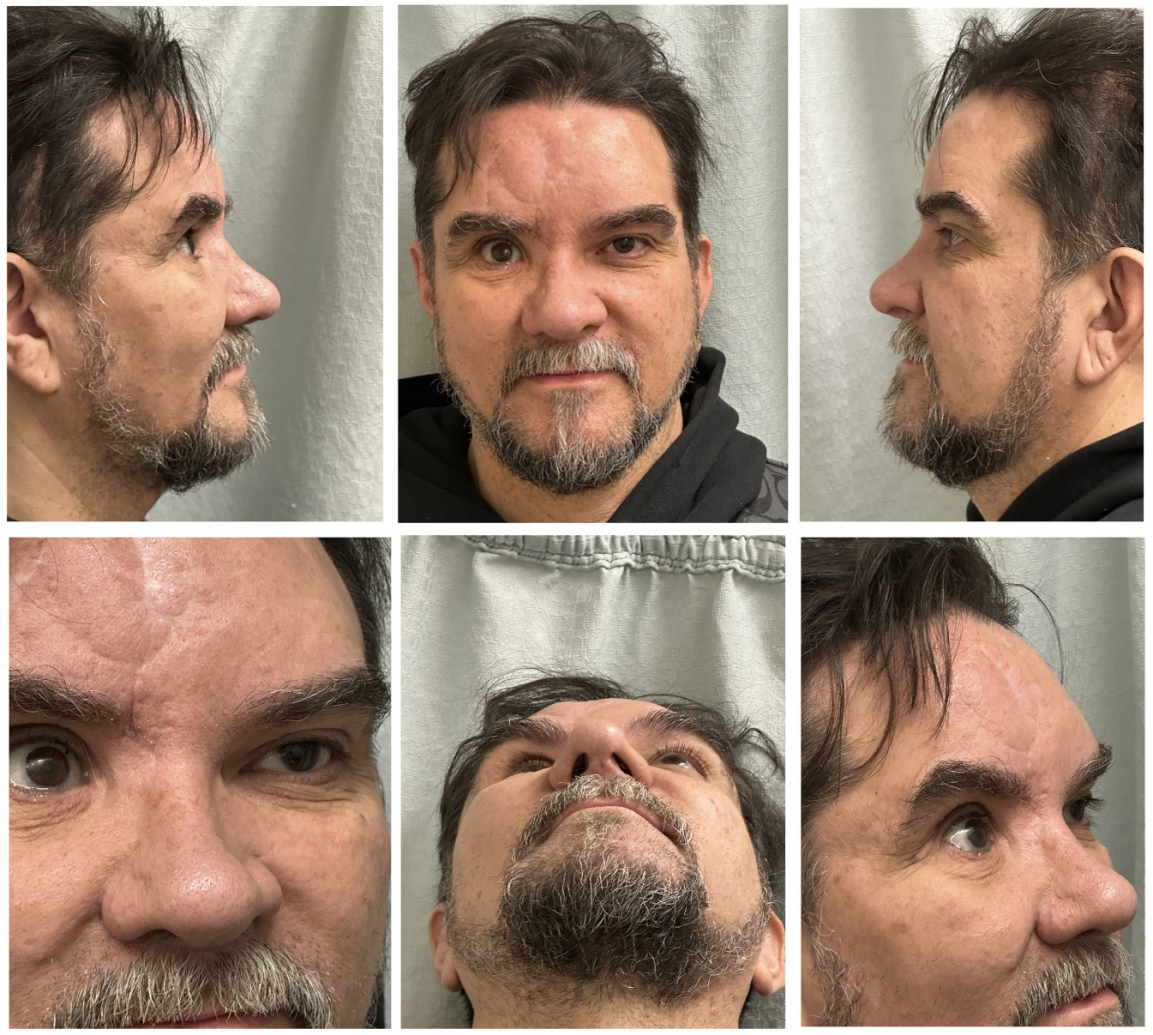
Postoperative images after recovery.

A limited superomedial orbital osteotomy was performed, enabling segment mobilization and repositioning to restore the orbital contour where prior reduction of fragments was insufficient. Despite this, a deficit remained in the skeletal support of the superomedial orbital rim. Further orbital reconstruction was avoided to preserve the existing right globe prosthesis. Therefore, to improve contour and cosmesis, we elected for soft tissue augmentation using a 30 × 8 mm moldable allograft made of 0.5 mm diced costal cartilage mixed with fibrin glue (Figure 3a). Also, the frontal bone irregularity was improved using a Medpor Titan implant (Figure 3b). An open rhinoplasty was then performed, while extending the orbital rim construct into the radix augmentation. The molded and trimmed graft was shaped to fit the radix contour, extended laterally toward the medial orbital rim, blending between the radix and the orbital rim for a seamless transition. The dorsal graft was subsequently blended to create a continuous natural transition from the radix to the nasal tip. To provide long-term tip support and projection, a septal extension graft coupled with intradomal and interdomal tip suturing techniques was used to shape the dome region of lower lateral cartilage and refine the nasal tip (Figure 3c). The procedure did not have any complications, and the postoperative pictures (Figure 4) were obtained 6 weeks after the procedure.

Over the last decade, DCGG has emerged as a powerful tool routinely used for reconstruction and/or augmentation of the nasal dorsum and radix.^1–3^ Mixing the solutions of Tissel (Baxter International Inc.) converts fibrinogen to fibrin and forms a sealant clot.^4^ Diced slivers of cartilage with fibrin become pliable and can be structured into the appropriate size and shape prior to insertion. Studies have demonstrated that the fibrin sealant enhances the proliferation and migration of human chondrocytes via secretion of chondrocyte-specific extracellular matrix components.^5,6^ Furthermore, sonographic morphometry on DCGG recipients showed overall graft stability at a 15-month follow-up, while histological evaluation at 20-month follow-up revealed vital cartilage with signs of regeneration.^3^

Orbital fractures present in 10%–25% of injuries from craniomaxillofacial trauma, including zygomatic-orbital fractures, blow-out, and panfacial fractures.^7,8^ Cartilage grafts offer low absorption rates with low risk of rejection and infection.^1^

Adapting DCGG onto orbital rim reconstruction extends its adaptability, smooth contouring ability, and reduces the risk of warping. Thus, an existing technique was adapted to achieve superior orbital rim reconstruction with a smooth, aesthetically seamless contour. In our case, post recovery, the patient was content with his appearance, and no further complications were noted.
